# Properties of Concrete Paving Blocks and Hollow Tiles with Recycled Aggregate from Construction and Demolition Wastes

**DOI:** 10.3390/ma10121374

**Published:** 2017-11-30

**Authors:** Carlos Rodríguez, Isabel Miñano, Miguel Ángel Aguilar, José Marcos Ortega, Carlos Parra, Isidro Sánchez

**Affiliations:** 1 Department of Construction Materials, Centro Tecnológico de la Construcción, Polg. Oeste, 30820 Alcantarilla, Spain; crodriguez@ctcon-rm.com (C.R.); isabelminano@hotmail.com (I.M.); 2 VIPRELUC, Ctra A-318 Puente Genil Lucena, km. 23’65 Apdo C. 195, 14500 Puente Genil, Córdoba, Spain; miguelangel.aguilar@vipreluc.com; 3 Departamento de Ingeniería Civil, Universidad de Alicante, Ap. Correos 99, 03080 Alacant/Alicante, Spain; jm.ortega@ua.es; 4 Department of Architecture and Building Technologies, Technical/Polytechnic University of Cartagena, Paseo Alfonso XIII, 30203 Cartagena, Spain; carlos.parra@upct.es

**Keywords:** mixed recycled aggregate, concrete recycled aggregate, ceramic recycled aggregate, non-structural concrete, precast concrete

## Abstract

In recent years there has been an increasing tendency to recycle the wastes generated by building companies in the construction industry, demolition wastes being the most important in terms of volume. The aim of this work is to study the possibility of using recycled aggregates from construction and demolition wastes in the preparation of precast non-structural concretes. To that purpose, two different percentages (15% and 30%) of natural aggregates were substituted by recycled aggregates in the manufacture of paving blocks and hollow tiles. Dosages used by the company have not been changed by the introduction of recycled aggregate. Precast elements have been tested by means of compressive and flexural strength, water absorption, density, abrasion, and slipping resistance. The results obtained show the possibility of using these wastes at an industrial scale, satisfying the requirements of the Spanish standards for these elements.

## 1. Introduction

The recycling and reusing seems to be more and more a necessity in our society. The most polluting sectors should be the ones more concerned about this tendency. In recent years, the Spanish construction industry has generated very large amounts of construction and demolition waste (C&DW) that is mainly stored in dumps. Directive 2008/98/CE of the European Parliament [1] established the necessity of reducing the natural resources consumption, and the need of recycling. It was established with the aim of reusing, recycling, and giving value to 70% of the C&DW generated by 2020. Even though the objective was clearly stated, nowadays in Spain only about 15% (10% in 2013, as stated by Mália et al. [2]). The construction and demolition wastes are about 25–30% of the total wastes generated in the country [3]. There is a clear requirement to improve the recycling percentage and reach values to a similar level to those of other European countries, such as Holland, Belgium, or Denmark, where about 80% of the waste is recycled, and it is necessary to use this waste on a massive scale, otherwise the effort will have a minor effect on the recycling measures [4].

Recycled aggregates are obtained after C&DW processing. Depending on their origin, the recycled aggregates can be classified as asphalt, ceramic, concrete, or recycled mixed aggregates (RMA). The RMA is about 80% of the C&DW [5] and include a great variety of materials, such as those just mentioned and, in minor proportions, plaster, glass, plastic, and so on. In the direction of having a more sustainable sector, the Spanish standard for concrete [6] states, and promotes, the use of recycled aggregates for the preparation of concrete, both for structural and non-structural uses. It allows the use of coarse recycled aggregates coming from concrete up to a percentage of 100%. The standard considers that a 20% replacement of coarse aggregate will cause no loss of properties. However, that standard allows the use of recycled aggregates in greater rates and with no restriction to the aggregate type for non-structural uses, due to the lower strength required in these elements.

Previous results have shown that the use or RMA causes a decrease of the compressive and flexural resistances (among 10–30% for a 50% replacement rate), increases the porosity (around 26% increase for a 75% of RMA), and the water absorption of concrete (about 50% increase for 50% recycled aggregate) [7,8,9] of the manufactured elements with an aggregate composition of 44.20% mortar, 18.30% concrete, 35.60% red ceramics, 0.1% white ceramics, and 1.8% rocks. However, some of these elements fulfil the requirements of the standard, for example in [7] kerbstones prepared with 25% of recycled aggregate have a resistance greater than 3.5 MPa, as required in the standard, and are below the 6% of water absorption up to a 75% of RMA used to replace natural aggregates.

There is another way of making the construction industry more sustainable, and it is by using cements with low clinker content [10], by means of using mineral admixtures such as blast-furnace slag CEM III B [11,12], fly ash, CEM II B- [11,13] or silica fume, and CEM II A-D [14,15]. All these mineral additions are industrial wastes, and among them the one that improves the properties of concrete at low levels of admixture is silica fume. With 15% of silica fume, referring to the weight of cement, the mechanical strength increases in a significant way, from approximately 63 to 82 MPa at 28 days [16] and it also decreases the water sorptivity, about 2.3 m^3^ × 10^−7^/(min)^1/2^ [17] due to the more compact structure that is developed due to the pozzolanic reaction of silica fume with the portlandite produced in the hydration of the calcium silicates [18]. The good properties of this addition have led Spanish authorities to recommend it for high-performance concrete in the standard [6].

There are several works that try to improve the properties of the elements prepared with recycled aggregates. On one hand, some authors try to use cements with additions to provide a higher resistance and impermeability to the elements. It has been shown that the incorporation of high calcium fly ash improves the mechanical behaviour of the precast elements, around 12% after 400 days [19], and the use of standard fly ash improves mechanical resistance 10% for a 25% replacement of cement by fly ash, as well as the resistance to carbonation (0.2 mm/month^1/2^) and chloride ingress (about 14%) in precast elements [20]. The other way of improving the properties that has already been tested is the use of different compositions. Instead of using the habitual Bolomey methodology. In [21] a methodology was established that took into account the nature of the recycled concrete aggregate, and it improved the results obtained, at least at the laboratory scale, in terms of water penetration, from 30 mm to 10 mm, and chloride ingress from 24.4 mm to 22.1 mm. As a general fact, it is established that the use of coarse recycled aggregate under certain quality control procedures, the same procedures as for natural aggregates, and at rate of substitution of ca. 50%, and for some applications even 100%, could be used to obtain structural concrete [22] with good durability properties, including corrosion phenomena [23], and also in high-performance concrete [24].

The study of the effect of including recycled sand has been pursued more recently, as was stated by Neno [25]. In this work the possibility of using up to 20% of recycled sand to produce construction mortar was proved with no risk to the integrity, from the point of view of water absorption of concrete, mechanical strength and water vapour permeability. The shrinkage shown by mortars containing fine recycled aggregate was almost double as compared to mortars with natural aggregate. This increase of the shrinkage is possibly due to the high elasticity modulus. Some recent studies in this field show clearly poor performance of mortars with recycled concrete fine aggregate, in terms of both mechanical resistance and durability [26,27], but even though there is a mechanical strength loss some authors have proved that it is possible to use it in structural real-size elements under flexural bending [28] due to their energy dissipation ability. A 50% fraction of recycled fine aggregate was proved to be the maximum amount of recycled fine aggregate for masonry mortars for indoor use [29], even though the shrinkage of these materials was great compared to those with natural aggregates. This paper uses the same idea of Fernandez-Ledesma, but the difference is that materials in [29] were prepared in the laboratory. In this work, with the intention of transferring knowledge to the industry, the construction elements have been manufactured in companies under real production conditions.

The present study tries to provide a solution to the problem generated by the demolition wastes and their treatment prior to the possibility of recycling the waste. This is done by means of manufacturing two very useful precast products: paving stone and hollow tiles. Other studies have been conducted with kerbstones, paving blocks, floor blocks, bricks, and blocks [8,30,31,32]. In order to study how recycled aggregates affects the properties of these elements, different substitution rates have been used, testing the influence of the percentage rate in the resistance, in bending strength, water absorption, density, abrasion, and slipping resistance. 

In order to improve the mechanical strength, water absorption, abrasive resistance, and slipping resistance prepared silica fume has been used. There are some papers that explore the use of recycled concrete aggregates (RCA) and silica fume to improve the mechanical and durability properties of the products [33,34,35]. In other works the silica fume is used to produce concrete. This concrete, with good properties, is crushed and used as recycled aggregate [36]. Other authors impregnate the recycled aggregate concrete with silica fume [37]. In both cases the results are good as compared with the original concrete.

One of the main problems of using RMA is the differences that can be found in their composition. The analysis of the aggregates used in [7,8,38] shows the following average composition in terms of main components: unbound aggregates: 34.6 ± 33.9; concrete: 49.7 ± 35.8; and ceramics (mainly red): 12.9 ± 5.0. The high standard deviations reflect the very large differences among different RMA, however, with different types of precast elements, or mass concrete samples. Sousa et al. [38] prepared concrete blocks using an RMA with 75% coming from concrete, 15% ceramic, and 10% from soil. The blocks prepared with 30% RMA showed an average loss of resistance of 44%, and an increase in water absorption of 47%. Kou et al. [38] prepared mass concrete, using two different, but similar, RMAs with an average composition of 76% concrete, 9.5% natural aggregates, and 13% ceramic material. Using 50% RMA on different samples, the compressive strength decreased around 18% (average), the shrinkage of concrete increased around 13%, and the resistance to chloride ingress increased about 32% at 28 days. More recent studies [39] have probed that it is possible to use a RMA with 47% concrete, 21.2% ceramics, and 26.3% unbound aggregate to dyke blocks by mixing 50% of the RMA with 50% of slag coarse aggregate. Other work [40] has produced hollow tiles by mixing concrete and brick wastes. The results obtained are in agreement with the literature, but it is shown that, for a 35% replacement, the decrease of the concentrated load test is of only 5%, making these elements accurate for constructive purposes. 

Most of these studies have been carried out under controlled laboratory conditions which, on many occasions, differ substantially from the conditions in real companies. In addition to that, under laboratory conditions the amount of recycled aggregate that can be used is not large enough to increase the amount of recycled C&D wastes. In order to increase the recycling rate (as a percentage of the total wastes generated) to achieve the European guidelines in a safe way, effort has to be made to study the properties of elements manufactured at industrial scale, in companies that usually produce this type of precast elements. 

In this work there are three main novelties: first, the use of silica fume to try to improve the properties of precast elements prepared with construction and demolition wastes; second, the change of the composition parameters for certain types of recycled aggregates; and, finally, the most important, the production at industrial scale of the elements, studying the behaviour of different recycled aggregate types. This aspect will be essential to recycle a large percentage of demolition wastes and reach the recycling rate compromised for 2020.

## 2. Materials

### 2.1. Cement

The cement type used was a CEM II/A-L 42,5 R according to the Spanish standard UNE-EN 197-1 [41] with an approximate clinker content of 84% and 15% of limestone. Its physical and chemical properties are summarized in Table 1.

The cement used includes a certain amount of limestone. This cement was chosen because, as some products used silica fume, it is the closest to the ordinary portland cement (OPC) that the Spanish standard forces to use with silica fume.

### 2.2. Addition

The addition that was used was silica fume. It was provided by Ferroatlantica (A Coruña, Spain) and was served densified. Its properties are summarized in Table 2. In the selected samples it was added a 6% of cement mass. The Spanish standard limits the amount of silica fume to 10% of the mass of cement. Six percent was selected to avoid making the products more expensive.

### 2.3. Natural Aggregate

**Figure 1:**
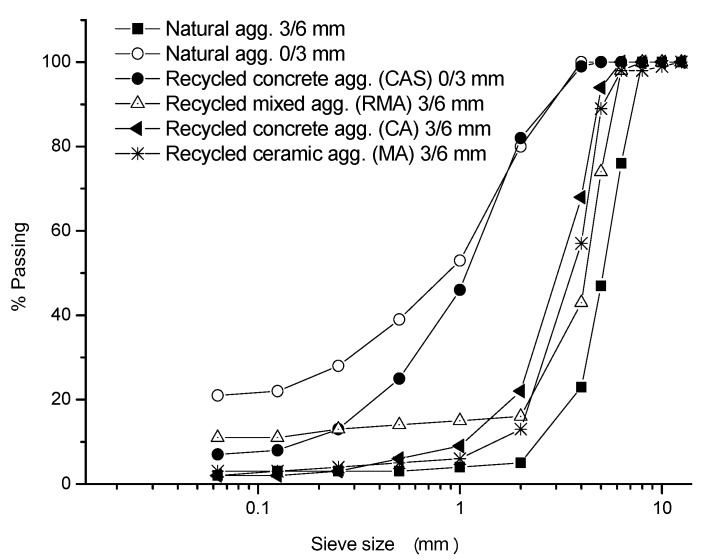
Grading curves of recycled and natural aggregates.

Table 3 shows the main properties of the natural limestone aggregates, produced in a quarry in Estepa, Sevilla (Spain). Both the fine (0/3) and medium (3/6) fractions were used to manufacture hollow tiles and paving blocks. The grading curve of natural aggregate can be seen in Figure 1.

### 2.4. Recycled Aggregate

In this work, three different types of recycled aggregates have been used. One of them was mainly formed by concrete wastes generated in a precast elements manufacturing company (Vipreluc, Córdoba, Spain). With these elements two different fractions of aggregate were produced: 0/3 mm (CAS) and 3/6 (CA). The second type was formed by ceramic wastes (MA), generated in a brick producing company, so the ceramic material was terracotta. The third, and last, type used was a recycled mixed aggregate (RMA) produced in a construction and demolition waste treatment plant. The recycled mixed aggregate contains other types of wastes, such as stone materials and asphalt in their composition. According to [42], the percent composition is: concrete, 36.8; masonry, 18; unbound aggregates, 14.2; gypsum, 13.4; asphalt, 8.8; floating particles 2.1, and other 4.8. Table 4 show the results of the physical characterization tests for each type of recycled aggregate and Figure 1 shows the results of the grain size analysis for every type of recycled aggregate.

Recycled aggregates were not pre-saturated because the experts at the manufacturing plants did not find it appropriate for industrial production. The total amount of water was adjusted as a function of the recycled aggregate type and percentage, to ensure the same amount of free water for the cement hydration reactions. Knowing the water absorption of the aggregates, it is easy to calculate the total water.

## 3. Experimental Program

### 3.1. Products and Dosages

Two different products have been prepared: pavement blocks and hollow tiles. 

**Figure 2:**
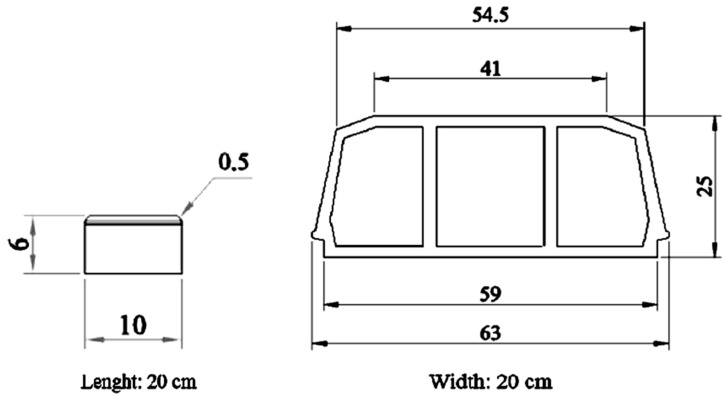
Geometrical dimensions (in cm) of pavement blocks (**left**) and hollow tiles (**right**) used in this study.

The pavement blocks and hollow tiles were manufactured with two different percentages of natural aggregates replaced by recycled aggregates: 15% and 30% of volume fractions. Pavement blocks and hollow tiles were also prepared using natural aggregates to have a reference of the conventional properties of these elements. The geometry used and element’s dimensions are shown in Figure 2.

As can be seen in Figure 2, in the case of paving blocks samples are formed by two different layers, one of the of thickness 5.5 cm, while the second layer is only 0.5 cm thick. Recycled aggregates are only used in the base layer (5.5 cm thickness) in the paving blocks. In the case of hollow tiles, recycled aggregates are used in the whole element. The thickness or the walls of the hollow tile (2 cm) was the limiting factor for the maximum size of the aggregates used, as it happens in the industry commonly.

**Figure 3:**
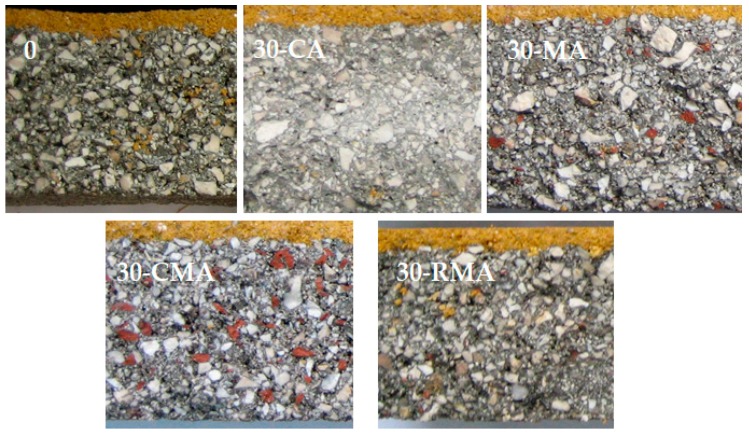
Paving blocks photographs for the different types of the recycled aggregates used.

Figure 3 shows the paving blocks manufactured using the different types of recycled aggregate used in this work.

The concrete composition for each element is shown in Table 5. The nomenclature used makes reference first to the type of non-structural precast element, paving blocks (PB), hollow tiles (HT), the percentage of recycled aggregate used, 0% (0), 15% (15), and 30% (30), the type of recycled aggregate used, recycled concrete sand (CAS), recycled concrete aggregate (CA), recycled masonry aggregate (MA), 50% recycled masonry and 50% concrete aggregate (CMA), recycled mixed aggregates (RMA), the dosage method based on the maximum compactness of the aggregates (C) and, finally, the content of silica fume (S).

For the fabrication of the paving blocks two different dosages were used: one for the fine recycled concrete aggregate, 0–3 mm (CAS) where it was used a methodology described in [43] to obtain the maximum compactness of the aggregates. The second one used the recycled fraction 3/6 mm, introducing no change in the usual dosage used by the company. For both series the corresponding reference concrete samples were produced, as well (PB_0_-C y PB_0_). In all of these products, the dosage used was the one commonly used in the manufacturing companies that have participated in this work.

The maximum compactness of the aggregates was achieved by Martinez Conesa et al. [43] by using a factorial experiment design and after compacting different mixes measuring their weight to establish a model based on the response surface method. This methodology combines different volumes of aggregates of different size ranges, and after compacting the aggregate mixture using 125 hits in the compacting device described in UNE-EN 196-1. The volume of holes is determined knowing the density and mass of the aggregates. The surface response methodology is used to calculate the mix of aggregates that gives the optimum compactness. 

To check the influence of silica fume in this type of precast elements (vibropressed with recycled aggregates) new elements were prepared for paving blocks manufactured with recycled masonry aggregate (MA) and recycled mixed aggregates (RMA), adding a 6% of silica fume referred to the weight of cement (PB_1_-MA-S, PB_2_-MA-S, PB_1_-RMA-S, PB_2_-RMA-S). Authors did not want companies to change their usual dosages in order to study the possibility of using, at the industrial scale, the results of the work. For that reason silica fume was added as if it was an extra element of the mixture.

The amount of water used for every dosage was modified to obtain the same value in the slump test as the concrete reference (0–1 cm). Once all samples were prepared they were sent to the curing areas where they were kept for 28 days until testing. Conditions in the curing area were room temperature and relative humidity between 75% and 80%.

### 3.2. Tests

In all the industrial settings, tests have been conducted to determine the consistency of concrete according to the UNE EN 12350-2 standard [44].

Resistance of pavement blocks and the results of the concentrated load test and flexural strength for hollow tiles were determined by tests at 28 and 90 days, according to the UNE EN 1338 [45] and UNE EN 15037-2 [46] standards, respectively. Seven samples were tested for the paving blocks, and six in the case of hollow tiles for every test performed.

Water absorption tests (of prepared concretes) were carried out in paving blocks at 28 and 90 days, and hollow tiles at 90 days according to the UNE EN 1338 standard (valid for both elements). The absorption values for four pavement blocks and hollow tiles by each mixture were recorded.

The resistance to abrasion and slipping were determined in pavement blocks at 90 days, following the procedure described in the UNE EN 1338. Wear resistance made by abrasion, as well as slipping resistance, were determined in the inner face where recycled aggregates had been used. The outer surface was not tested since RMA were not used in that part of the elements.

Concrete density was determined according to the standard UNE EN 12390-7 [47].

## 4. Results and Discussion

### 4.1. Compressive and Flexural Strength

**Figure 4:**
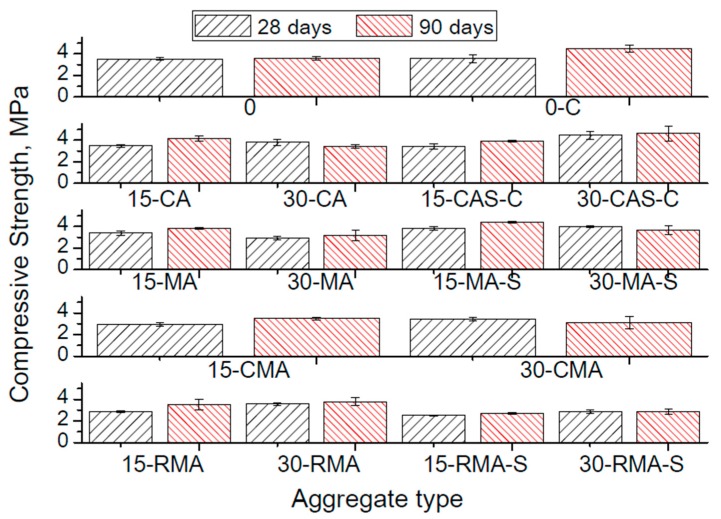
Results of mechanical strength in paving blocks, as a function of the aggregate type and dosaging method (see Table 5 for nomenclature).

Table 6 and Figure 4 show the results of the compressive strength for paving blocks. In Table 7 the results of the concentrated load test for hollow tiles are presented, and Table 8 includes the results of the flexural strength for hollow tiles. The presented values are the average of all the samples tested, as it was explained in the previous point, and includes the standard deviation.

As it could be expected, in most of the cases of recycled aggregates, the increase in the recycled fraction causes a decrease of mechanical resistance even though, in some cases, with the fine fraction of concrete aggregate (CAS), the results are similar, or even higher, than the reference material, as can be seen in Table 6.

Minimum values of compressive resistance, required by the standard UNE EN 1338 [45] on paving blocks are 3.5 MPa. There are only requirements for trading this products on their resistance. At 90 days all samples (taking into account the average value of the resistance), excepting PB30-CA, PB30-MA and PB 15 and 30-RMA-S would fulfil this requirement. It means that the silica fume is not working as it was expected, possibly due to the conditions in the industrial curing chambers. In this samples the effective water:cement ratio was lower leaving less water for the reaction of silica fume, making more difficult the development of its beneficious effect. The RMA could be used with total guarantees in this element up to 30%. Concrete and ceramic aggregates could be used up to 15% replacement in the present conditions. The standard deviations are quite high, but some elements prepared with recycled aggregate show higher resistances than the reference one, which is usually manufactured in the company with good results. Thus, it means that the rest of the products could also be used for construction purposes.

In the case of paving blocks (Table 6) the use of the recycled concrete fraction 0/3, in volume percentages of 30% of natural sand (especially PB_30_-CAS-C), gives paving blocks with similar compressive strength as the reference material obtained using the dosing technique to achieve the maximum compactness of the aggregate skeleton (PB_0_-C). If those results are compared with the strength of paving blocks prepared only replacing part of the coarse (3/6) aggregates (PB_15_-CA and PB_30_-CA), the obtained results are similar. This fact confirms that, up to a certain level, the replacement of natural aggregate does not significantly decrease the compressive strength, even though the elements are not prepared in a laboratory, but at the industrial scale. Some works have studied both the replacement by concrete recycled aggregate both in the coarse and fine fractions in vibropressed precast elements, and they reached the same conclusions, showing that up to 50–60% there is no significant influence of the use of concrete recycled aggregate on the compressive resistance [48,49], even though these works were conducted in a laboratory and not at the industrial scale. 

The use of a dosage method to optimize the compactness of the aggregate skeleton (PB_0_-C) is an accurate methodology to improve the compressive strength of paving blocks, since it increases the mechanical resistance about 25% after 90 days, as compared with the standard dosage (PB_0_) used by the company. It could be used to improve the resistance of the precast elements, even in the fine fraction, as is shown by the results of the elements of the series CAS-C, that give a larger compressive strength of all the elements tested, including the ones prepared with the optimized dosage of the coarse aggregate. This result is promising, and seems to open a way of using recycled aggregates just by optimizing the dosage of both coarse and fine fractions.

The replacement of a 15% of the coarse natural aggregate by all the recycled aggregates studied (CA, MA, CMA, and RMA) does not cause a significant loss of mechanical resistance after 90 days, compared to the reference paving blocks. If the volume of recycled aggregate changes to 30% there are differences depending on the composition of the recycled aggregates. Samples with concrete CA and mixed RMA aggregates give similar values to those of the reference after 90 days, while the use of masonry MA and concrete and masonry CMA aggregates cause a loss of compressive strength of about 11% and 14%, respectively. 

Even though the properties have not been studied at longer times, due to the high presence of gypsum (13.4%) in the RMA some problems could be expected due to the formation of ettringite. It would be necessary, in order to produce this type of precast elements at industrial scale with no risk of deterioration to keep the sulphate content in the allowed limits (<0.8%, referred to cement weight) by the EHE-08 [6].

As stated in previous sections, 6% silica fume (referring to cement weight) in prepared paving blocks has been included with MA and RMA. The reason of selecting these two types of aggregate is that they are the types more commonly obtained in building demolition, which can be obtained without a complex classification system. This fact implies a lower cost for the recycling of these aggregates, and increases the feasibility of their use at an industrial scale. The results obtained are contradictory. In the case of MA the resistance after 90 days improved about 10% as compared with the elements without silica fume. However, the use of silica fume together with RMA causes a loss of compressive strength of about 20% when compared to the elements without silica fume. These results could be attributed to two different reasons. The first are the poor curing conditions in the industrial process, which do not ensure the relative humidity of 100%, affecting the complete hydration of cement, and the pozzolanic reaction of silica fume. This effect has been observed for active additions with no hydraulic activity, such as class V fly ash, according to the Spanish standard [41]. For this type of addition a significant influence of the environment on the development of the pozzolanic reactions has been observed, a fact that causes a coarser pore network and worse service properties of the material [13,50]. On the other hand, the water absorption results of the aggregates, which will be presented later, show a greater water absorption by recycled masonry aggregates (MA, 16.7%) than by recycled mixed aggregates (RMA, 8.9%). The aggregates tend to absorb water during mixing, and this water could be realized later on, causing a “self-curing” effect [51] that has already been reported in the case of using RMA [7], improving then the properties of samples prepared with the aggregate with higher sorptivity. 

The minimum value of compressive resistance required by the standard is fulfilled after 90 days for most of the paving blocks prepared, except for PB_30_-CA, PB_30_-MA, PB_30_-CMA, PB_15_-RMA-S, and PB_30_-RMA-S. Results show that it is feasible to obtain paving blocks with 15% of recycled aggregate, no matter the source of that aggregate, fulfilling the requirements stated by the standard UNE EN 1338.

Regarding the two methods for improving the resistance of paving blocks (use of silica fume and optimization of the aggregate dosage) it has to be said that from our results when used in a real industrial production process the silica fume does not work properly, at least when used with recycled mixed aggregate (RMA), and this behaviour could be attributed to the lack of moisture in the industrial processes. This aspect (the relative humidity in curing chambers) should be taken more carefully to improve the resistance of the precast paving blocks due to the use of an active addition. In other work done in a laboratory, 15% fly ash was used substituting concrete and ceramic recycled aggregates and an increase of resistance was observed on the tested paving blocks [32]. In this case the samples were kept under immersion, facilitating all the hydration and/or pozzolanic processes.

**Figure 5:**
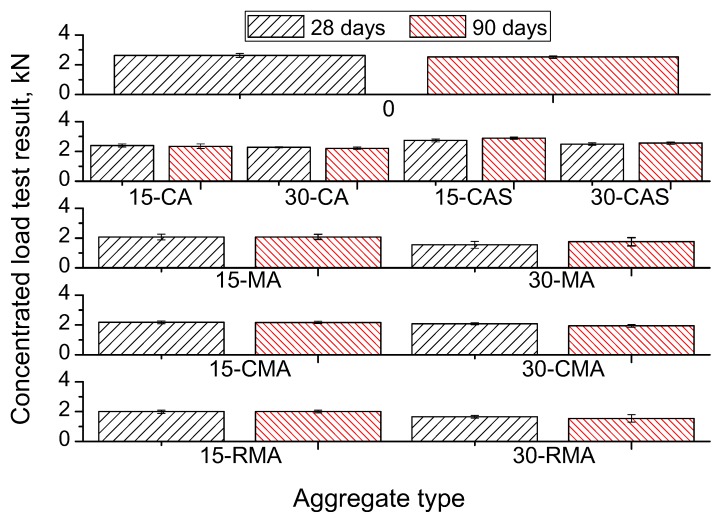
Results of the concentrated load test in hollow tiles, as a function of the aggregate type used.

The results of the hollow tiles show that an increase in the percentage of recycled aggregate causes strength loss for every type of recycled aggregate used in this study. The results of the concentrated load test are shown in Table 7 and Figure 5 while the results of the flexural strength are presented in Table 8.

In the case of using the recycled aggregate in the coarse fraction (3/6), for the concentrated load test (Table 7) there are clear differences as a function of the type of recycled aggregate used. The results of the concentrated load test show that the masonry aggregates (MA) cause a greater resistance loss, compared to concrete recycled aggregates (CA), as was observed in the case of the paving blocks. The strength lost is (at 90 days, for a replacement of 30%) 41% in the case of using MA, and 18% in the case of CA. These results confirm that the composition of the recycled aggregate has a big influence on the service properties of the concrete prepared, and it usually is due to the weakness of the ceramic material form masonry, compared with the material coming from concrete [49,52].

The use of the 0/3 recycled fraction, coming from concrete (CAS) improves the strength of the precast elements as compared with the results given by the 3/6 CA fraction. This result is very important because it shows that it is possible to obtain good quality precast elements using recycled sand. However, the results of elements prepared using recycled sand are not that good in other works [36]. Due to this difference the fine fraction cannot be discarded, but further in-depth research on the reason of this different behaviour are necessary to clarify the origin and to establish a procedure for using recycled sand.

The UNE EN 15037-2 standard [46] establishes that the minimum concentrated load resistance P_RK_ must be 1.5 kN for non-structural hollow tiles. Based on the results obtained in the study it has been calculated that the average minimum value for the concentrated load test strength P_N_ is 1.67 kN, calculated using the formula P_N_ ≥ P_RK_ + 1.48·σ [46], σ being the standard deviation. The results obtained show that, except for the dosage of HT_30_-RMA, all the manufactured elements containing recycled aggregates fulfil the requirement of the standard at 90 days, and most of them at 28 days.

If the evolution between 28 and 90 days of the values of the resistance of the hollow tiles is analysed there are no important differences among samples, due to the origin of the recycled aggregate. In another study, where the evolution of the strength of different elements was studied for up to one year [7], it was established that the evolution is slower in the long-term if recycled mixed aggregates are used.

**Figure 6:**
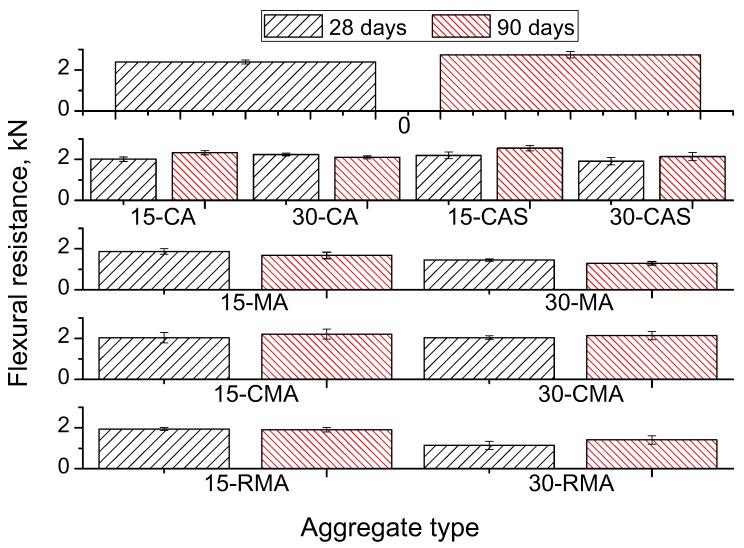
Results of flexural strength in hollow tiles.

An equivalent analysis could be done with the flexural strength of the hollow tiles, whose results are shown in Table 8 and Figure 6.

It can be seen in the tables and figures that the mechanical resistance of the precast elements decreases in some cases, from 28 to 90 days. In [7] where materials were also produced at the industrial scale, the resistance in some cases decreased. It has to be taken into account that the curing chambers of the companies do not have the same requirements as in the laboratory, and the care in the products’ manufacture is not the same. On the other hand, the water absorption of the aggregates is not instantaneous, and even though the water necessary for the absorption was given, it does not mean that it is goes straight to the aggregates and might cause drying, resulting in drying shrinkage, with the consequent loss of mechanical properties. The MA aggregate presents this anomaly more often and it could be related to their higher water absorption. 

### 4.2. Water Absorption

In general, the increase of the percentage of recycled aggregate produces an increase of the water absorption of the precast elements even though, in the case of the paving blocks, this result is not true for every set of samples manufactured. The results can be seen in Table 9 for paving blocks and in Table 10 for hollow tiles.

The use of the concrete recycled fine fraction (0–3 mm, CAS) produces, in general, hollow tiles with lower water absorption than when the coarse fraction from recycled concrete is used, both in the case of paving blocks and hollow tiles. The same conclusion can be drawn if the results of the fine fraction are compared with the coarse fraction of the rest of the recycled aggregates (MA, CMA, RMA) in hollow tiles. This result is coincident with the good results obtained of the mechanical properties of the precast elements. In that case the use of the fine fraction of recycled concrete aggregate also improved the mechanical properties of both paving blocks and hollow tiles (independent of the dosage technique used). Both results show that the fine fraction of recycled aggregate should not be rejected and discarded as a construction material, even though some works have different results as compared with this work [9,31]. As it was stated in the discussion of the mechanical properties of the precast elements, more research is necessary to clarify the reasons of the different behaviour of the fine fraction in different studies.

A comparison of the results of the paving blocks prepared using the coarse fraction as recycled aggregate show that the masonry aggregate is the element that increases the water absorption more (with 15% of recycled aggregate, an increase of 4%, and with 30% of MA, an increase of 14%, compared with the absorption of the reference, and 21% and 57%, respectively, for the hollow tiles). Recycled aggregates coming from concrete (CA) in the coarse fraction are the ones that produce a lower increase of the water absorption (−1.6% and −3.3% at 90 days for paving blocks, and 12% and 23% for hollow tiles). As can be seen, the results for paving blocks are very similar, or a slight improvement on the results of the reference concrete. The recycled aggregate with the lower water absorption was CA with 3.9%, while the absorption was 17.6% for MA, being much more porous, in general. These values can justify the differences in the absorption of the precast elements. 

As can be seen in the tables, the use of CMA aggregates increase the water absorption as compared with the CA results. CMA should also have a higher absorption of water, due to the presence of ceramic aggregates. This result confirms that including masonry wastes as recycled aggregates, that are mainly ceramic, increases the water absorption of the precast elements as it was stated in other work [52]. These results also are in agreement with the results of mechanical properties of both precast elements, because the resistance decreased as the masonry aggregate was included, possibly due to a higher porosity. This result also confirms the different behaviour of the addition of silica fume to different precast elements. The greater water absorption of concrete, clearly due to the presence of masonry aggregate, acts as a water reservoir, which will help the development of the pozzolanic reactions of the silica fume, and the strength gain in the elements prepared with CMA.

The results of the elements manufactured with RMA show a similar behaviour to the reference concrete in the case of paving blocks, slightly better for the case of 30% of RMA, but for the hollow tiles produce an increase in water absorption of 27% and 40% for 15%, and 30% of natural aggregate substitution by RMA. The water absorption of the RMA was 8.9, slightly closer to that of the CA, which is why the RMA causes intermediate water absorption among the CA and MA. 

The effect of silica fume does not decrease the water absorption in whichever type of recycled aggregate is used (MA or RMA). In fact, in the case of using RMA, it increases the absorption of water by about 40%. This fact, together with the evolution of the mechanical resistance does not make the use of silica fume suitable to improve the results of these elements with recycled aggregates, possibly due to the industrial curing conditions.

Water absorption in paving blocks is related to their climatic resistance. According to the UNE EN 1338 [45] standard regardig pavement blocks, all concretes will be tagged as 1. It has to be pointed out that the requirement of some climatic resistance for pavement blocks is a choice of the country where the standard is used. 

There are surprising data, such as that of the PB0-C samples, which are supposed to be more compact and have a greater water absorption as compared with PB0 samples or that, at 90 days, PB30-CA and PB-30-CAS present lower water absorption as compared to the elements with the same type of aggregate, but only at 15%. The same fact can be found for HR-15-CMA and HT-30-CMA. This fact might be due to the compacting deficiencies or simply to the non-optimal conditions in the industrial processes. However, since the standard does not apply any requirement to this elements. Further research is necessary following the behaviour of the elements under service conditions and in the long-term. 

### 4.3. Density

As could be expected from the literature review [53,54] the density of both paving blocks and hollow tiles decreases as the percentage of substitution of limestone natural aggregate is replaced by recycled aggregate, MA, CMA, and RMA due to the lower porosity of the recycled aggregates compared to the natural aggregates. The results of the density are shown in Table 11. As can be seen in the table, the use of concrete recycled aggregate both in the coarse (CA) or fine (CAS) fraction does not produce a significant change in the density of the produced elements. This fact is due to the low difference in density of the natural recycled aggregates, as well as the percentages of substitution used, to a maximum of 30%.

These results are in keeping with the rest of results presented. The MA gives the precast product the lower density, meaning the higher water absorption and the lower mechanical strength in the case of paving blocks.

A difference in the density of the paving blocks and the hollow tiles can also be seen, being lower than the density of the tiles. This fact is also coincident with the results of water absorption, could be due to the difficulty in compacting this type of elements, due to its geometry, which is shown in Figure 2.

### 4.4. Abrasive Resistance

The results of the abrasive resistance of the paving blocks are shown in Table 6. This test has no sense in hollow tiles, since those elements are not intended to suffer wear due to traffic or some other event, as in the case of paving blocks. It has to be pointed out here that the abrasive resistance was tested on the lower face of the elements, because this face is the one containing recycled aggregates, and that fact can justify the low values of the abrasive resistance obtained.

The abrasive resistance of paving blocks is similar to the reference ones when the recycled aggregate is incorporated. The use of the coarse fraction of MA and RMA causes a decrease in the abrasive resistance among 7.5 and 10 mm, independent of the type of aggregate and the percentage used. Other researchers had found that the abrasive resistance was only modified when the percentage or recycled aggregate was higher than 40% [55]. In that paper the presence of ceramics and unbound aggregates was higher than the RMA used in this study, while the concrete particles remained in approximately the same range (36.8%). The good behaviour shown by the elements prepared with ceramic aggregate (CA) with respect of abrasive resistance indicates that those aggregates have great importance in the abrasive resistance of the precast elements. This difference demonstrates the importance of the composition of the recycled aggregate in the behaviour of the products manufactured as it has already been shown [7], especially in the case of the presence of ceramic materials.

This fact is confirmed by the results of the CMA (50% CA + 50% MA). In this case the abrasion resistance only decreases 4.8 mm for a 15% of substitution and 6.8 mm for a substitution of 30%. The ceramic material is clearly weaker than the concrete aggregate from the point of view of abrasion.

The use of silica fume improves the behaviour against abrasion when used with the MA and RMA aggregates. It allows obtaining results similar to the reference concrete excepting in the case of using 30% of RMA. In this case results do not improve as a consequence of using silica fume. More research is necessary on the role of silica fume, but it should be focused on improving the mechanical behaviour and water absorption of precast elements.

Finally, the dosage searching the maximum compactness of the aggregates does not present significant differences with the reference one.

Again, some unexpected results in the properties arise, as the density of the PB-15-RMA-S is lower than the density of the samples including 30% RMA. This value could indicate problems in the compaction. These results are in agreement with those of water absorption. The same comment about the need of further research under service conditions could be made here. 

### 4.5. Slipping Resistance

Paving blocks with recycled aggregates do not show important differences compared to the reference ones, independent of the aggregate type and the percentage used (see Table 12). This confirms an already presented result: the use of recycled aggregate does not influence the slipping resistance [7]. The same result has been obtained using concrete and glass wastes [56] and, in other work where only concrete recycled aggregates were used, this property was improved [49] as the percentage of recycled aggregate increased.

The dosage that maximizes the compactness of the aggregates does not show differences with the reference one.

## 5. Conclusions 

In accordance with this experimental study and the results obtained, the following conclusions can be reached:

- In general, the increase of recycled aggregate ratio causes a decrease of mechanical resistance. The composition of the recycled aggregate has a strong influence on the properties of the precast elements. The higher percentage of ceramic aggregate from bricks causes greater resistance loss, according to the literature, due to the weakness of the ceramic material.
- Paving blocks, with little exceptions, fulfil all the requirements established by the UNE EN 1338 standard. In spite of the big standard deviation present in the data, since most of them present values greater than the reference materials they could be used for construction. Samples with RMA could be used with all guarantees.
- In hollow tiles every sample studied satisfies the standard UNE EN 15037-2 except for the hollow tiles with 30% of RMA at 90 days.
- As a general fact, the increase of the percentage of recycled aggregate causes an increase in the water absorption of the prepared concrete samples. The presence of ceramic materials are especially important for this increase, due to the higher porosity of these materials. Some materials do not follow this rule, possibly due to compaction deficiencies.
- In the experimental setup used in the companies collaborating in the work, the use of silica fume seems to be not as effective as could be expected. It does not improve significantly the mechanical properties and the water absorption of the precast elements, possibly due to the curing conditions commonly used in the industry, or the lower effective w:c ratio used, that would leave less water available for the silica fume pozzolanic reaction.
- The density of paving blocks and hollow tiles decreases as the percentage of MA, CMA, and RMA increases, due to its lower density as compared to the natural aggregate used in the study. The unexpected results found in some samples reinforce the idea of a lack of compaction in the prepared samples.
- The use of the fine fraction of the concrete recycled aggregate 0/3 CAS shows the same behaviour as the coarse fraction 3/6 CA for all the studied properties.
- Slipping resistance of recycled concretes does not present considerable differences in relation with the slipping resistance of reference concretes.
- Abrasive resistance if paving blocks prepared with recycled concrete aggregates is similar to the reference ones. The rest of the recycled aggregates studied (MA, CMA, RMA) cause a decrease of the abrasion resistance, even though with the incorporation of silica fume this difference can disappear. Ceramic aggregate seems to have a positive effect in the abrasive wear resistance of the elements prepared in this work.
- Under the studied conditions it is possible to manufacture at industrial scale precast elements with RMA, CA, and MA. The high water absorption, which is not limited by the standard, would require further research of the materials under service conditions.

## Figures and Tables

**Table 1:** Physical and chemical properties of cement.

Parameter		Result
Composition	Clinker	84%
	Limestone (addition)	15%
Physical Prescriptions	Initial setting time	144 min
	Final setting time	190 min
Compressive Strength	Two days	30.2 MPa
	28 days	50.3 MPa
Chemical Prescriptions	Sulfur trioxide	3.09%
	Chlorides	0.01%

**Table 2:** Physical and chemical properties of silica fume.

Parameter		Result
Composition	% SiO_2_	92.00
	% C	2.00
	% Cl^−^	0.03
	% SO_3_	0.43
	% CaO	0.43
	% K_2_O	0.54
	% Na_2_O	0.25
	% Alkalis as Na_2_O	0.61
	% Al_2_O_3_	0.57
	% MgO	0.64
	% Elemental Si	0.18
Physical properties	Specific surface (m^2^/g)	26.40
	Apparentdensity (t/m^3^)	0.58
	Loss on ignition	2.10

**Table 3:** Properties of natural aggregate.

Properties	Aggregate 0/3 mm	Aggregate 3/6 mm
Water absorption (%) (UNE-EN 1097-6)	2.5	2.96
Density (g/cm^3^) (UNE-EN 1097-3)	2.74	2.6
Resistance to fragmentation (Los Angeles, UNE-EN 1097-2)	30	30
Fines (<0.063 mm) (%) (UNE-EN 933:10)	21	2

**Table 4:** Results recycled aggregates physical properties.

Parameter	Concrete Aggregate (CAS)	Concrete Aggregate (CA)	Masonry Aggregate (MA)	Recycled Mixed Aggregates (RMA)
	0/3 mm	3/6 mm	3/6 mm	3/6 mm
Water absorption (%)	4.6	3.9	17.6	8.9
Dry surface density (g/cm^3^)	2.39	2.48	1.87	2.15
Soluble sulfates (%) (UNE 103201)	0.60	0	0	2.5
Fines (<0.063 mm) (%)	7	2	3	11

**Table 5:** Dosages used for each element.

Mixture	Cement (kg/m^3^)	Effective Water (kg/m^3^)	Total Water (kg/m^3^)	Silica Fume (kg/m^3^)	Effective Water: Cement Ratio	Natural Aggregates 3/6 (kg/m^3^)	Natural Aggregates 0/3 (kg/m^3^)	Recycled Aggregate 3/6 (kg/m^3^)	Recycled Aggregate 0/3 (kg/m^3^)
Pavement blocks with recycled concrete sand (CAS)									
PB_0_-C	320	144	144		0.45	506	1600		
PB_15_-CAS-C	320	144	147.6		0.45	506	1359		209
PB_30_-CAS-C	320	144	151.3		0.45	506	1120		419
Hollow tiles with recycled concrete sand (CAS)									
HT_0_	230	104	104		0.45	1101	1160		
HT_15_-CAS	230	104	106.6		0.45	1101	986		152
HT_30_-CAS	230	104	108.7		0.45	1101	813		305
Paving blocks (PB)									
PB_0_	320	144	144		0.45	1012	1066		
With recycled concrete aggregate (CA)									
PB_15_-CA	320	144	145.2		0.45	860	1066	145	
PB_30_-CA	320	144	146.2		0.45	708	1066	288	
With recycled masonry aggregate (MA)									
PB_15_-MA	320	144	158.7		0.45	860	1066	109	
PB_30_-MA	320	144	173.4		0.45	708	1066	218	
PB_15_-MA-S	320	144	167.4	19.2	0.42	853	1057	108	
PB_30_-MA-S	320	144	182.1	19.2	0.42	702	1057	216	
With recycled 50% masonry and 50% concrete aggregate (CMA)									
PB_15_-CMA	320	144	152		0.45	860	1066	127	
PB_30_-CMA	320	144	159.8		0.45	708	1066	253	
With recycled mixed aggregates (RMA)									
PB_15_-RMA	320	144	150.6		0.45	860	1066	125	
PB_30_-RMA	320	144	157.3		0.45	708	1066	250	
PB_15_-RMA-S	320	144	159.3	19.2	0.42	853	1057	124	
PB_30_-RMA-S	320	144	166	19.2	0.42	702	1057	248	
Hollow tiles (HT)									
HT_0_	230	104	104		0.45	1101	1160		
With recycled concrete aggregate (CA)									
HT_15_-CA	230	104	105.2		0.45	935	1160	157	
HT_30_-CA	230	104	106.4		0.45	770	1160	314	
With recycled masonry aggregate (MA)									
HT_15_-MA	230	104	120.1		0.45	935	1160	119	
HT_30_-MA	230	104	136.1		0.45	770	1160	238	
With recycled 50% masonry and 50% concrete aggregate (CMA)									
HT_15_-CMA	230	104	112.6		0.45	935	1160	138	
HT_30_-CMA	230	104	121.25		0.45	770	1160	276	
With recycled mixed aggregates (RMA)									
HT_15_-RMA	230	104	111.2		0.45	935	1160	136	
HT_30_-RMA	230	104	118.4		0.45	770	1160	272	

The values shown for the recycled aggregates stand for the percentage of volume substituted.

**Table 6:** Results of compressive strength, MPa, of paving blocks, as a function of the aggregate type, dosaging method (see Table 5 for nomenclature), and time.

Age	PB0	PB_0_-C	PB_15_-CA	PB_30_-CA	PB_15_-CAS-C	PB_30_-CAS-C	PB_15_-MA	PB_30_-MA
28 days	3.52 ± 0.14	3.56 ± 0.36	3.48 ± 0.11	3.79 ± 0.25	3.42 ± 0.25	4.45 ± 0.34	3.39 ± 0.19	2.96 ± 0.16
90 days	3.60 ± 0.18	4.52 ± 0.32	4.18 ± 0.26	3.41 ± 0.15	3.90 ± 0.11	4.61 ± 0.68	3.86 ± 0.08	3.20 ± 0.50
	PB_15_-MA-S	PB_30_-MA-S	PB_15_-CMA	PB_30_-CMA	PB_15_-RMA	PB_30_-RMA	PB_15_-RMA-S	PB_30-_RMA-S
28 days	3.84 ± 0.18	4.01 ± 0.09	2.98 ± 0.16	3.41 ± 0.15	2.85 ± 0.08	3.58 ± 0.11	2.51 ± 0.06	2.87 ± 0.17
90 days	4.37 ± 0.08	3.65 ± 0.39	3.50 ± 0.12	3.10 ± 0.58	3.50 ± 0.48	3.79 ± 0.39	2.70 ± 0.09	2.87 ± 0.24

**Table 7:** Results of concentrated load test (kN), of hollow tiles, as a function of the aggregate type, dosaging method (see Table 5 for nomenclature), and time.

Age	HT_0_	HT_15_-CA	HT_30_-CA	HT_15_-CAS	HT_30_-CAS	
28 days	2.63 ± 0.13	2.41 ± 0.11	2.28 ± 0.03	2.74 ± 0.10	2.50 ± 0.08	
90 days	2.52 ± 0.08	2.35 ± 0.16	2.21 ± 0.09	2.89 ± 0.07	2.56 ± 0.08	
	HT_15_-MA	HT_30_-MA	HT_15_-CMA	HT_30_-CMA	HT_15_-RMA	HT_30_-RMA
28 days	2.08 ± 0.20	1.55 ± 0.23	2.18 ± 0.10	2.08 ± 0.06	2.00 ± 0.11	1.66 ± 0.09
90 days	2.08 ± 0.17	1.75 ± 0.27	2.17 ± 0.08	1.94 ± 0.10	2.01 ± 0.10	1.54 ± 0.25

**Table 8:** Results of flexural resistance (kN), of hollow tiles, as a function of the aggregate type, dosaging method (see Table 5 for nomenclature), and time.

Age	HT_0_	HT_15_-CA	HT_30_-CA	HT_15_-CAS	HT_30_-CAS	
28 days	2.39 ± 0.10	2.01 ± 0.11	2.24 ± 0.07	2.20 ± 0.16	1.91 ± 0.18	
90 days	2.74 ± 0.16	2.33 ± 0.10	2.10 ± 0.07	2.55 ± 0.13	2.14 ± 0.20	
	HT_15_-MA	HT_30_-MA	HT_15_-CMA	HT_30_-CMA	HT_15_-RMA	HT_30_-RMA
28 days	1.87 ± 0.14	1.45 ± 0.06	2.04 ± 0.26	2.04 ± 0.08	1.94 ± 0.07	1.14 ± 0.20
90 days	1.68 ± 0.15	1.29 ± 0.08	2.21 ± 0.24	2.14 ± 0.20	1.90 ± 0.11	1.41 ± 0.21

**Table 9:** Results of water absorption (%), of paving blocks, as a function of the aggregate type, dosaging method (see Table 5 for nomenclature), and time.

Age	PB0	PB_0_-C	PB_15_-CA	PB_30_-CA	PB_15_-CAS-C	PB_30_-CAS-C	PB_15_-MA	PB_30_-MA
28 days	6.7	7.6	6.7	6.9	6.6	6.9	7.1	7.6
90 days	6.1	6.6	6.0	5.9	6.6	5.9	6.3	7.0
	PB_15_-MA-S	PB_30_-MA-S	PB_15_-CMA	PB_30_-CMA	PB_15_-RMA	PB_30_-RMA	PB_15_-RMA-S	PB_30-_RMA-S
28 days	7.2	7.9	7.6	7.3	7.3	6.6	10.0	9.5
90 days	7.0	7.2	7.0	6.4	6.2	5.8	8.8	9.1

**Table 10:** Results of water absorption at 90 days of hollow tiles, as a function of the aggregate type, dosaging method (see Table 6 for nomenclature).

Age	HT_0_	HT_15_-CA	HT_30_-CA	HT_15_-CAS	HT_30_-CAS	
90 days	6.6	7.4	8.1	6.9	7.5	
	HT_15_-MA	HT_30_-MA	HT_15_-CMA	HT_30_-CMA	HT_15_-RMA	HT_30_-RMA
90 days	8	10.3	8.5	8	8.3	9.2

**Table 11:** Results of density at 28 days for paving blocks and hollow tiles, as a function of the aggregate type, and dosaging method (see Table 5 for nomenclature).

Paving Blocks								
Sample	PB_0_	PB_0_-C	PB_15_-CA	PB_30_-CA	PB_15_-CAS-C	PB_30_-CAS-C	PB_15_-MA	PB_130_-MA
Density, g/cm^3^	2.1	2.03	2.11	2.1	2.01	2.04	1.99	1.99
Sample	PB_15_-MA-S	PB_30_-MA-S	PB_15_-CMA	PB_30_-CMA	PB_15_-RMA	PB_30_-RMA	PB_15_-RMA-S	PB_30-_RMA-S
Density, g/cm^3^	2	1.95	2.03	2.05	2.04	2.06	2.01	2.03
Hollow tiles								
Sample	HT0	HT15-CA	HT30-CA	HT15-CAS-C	HT30-CAS-C	HT15-MA	HT30-MA	
Density, g/cm^3^	2.05	2.03	2.07	2.02	2.03	1.9	1.87	
Sample	15-CMA	30-CMA	15-RMA	30-RMA				
Density, g/cm^3^	1.97	1.99	1.95	1.98				

**Table 12:** Slipping and abrasive resistance after 90 days.

Mixture	Slipping Resistance	Abrasive Wear (mm)
PB_0_	90	20
PB_0_-C	93	23.5
PB_15_-CAS-C	96	23.3
PB_30_-CAS-C	87	20.5
PB_15_-CA	91	23.8
PB_30_-CA	89	20.8
PB_15_-MA	90	30.5
PB_30_-MA	91	27.5
PB_15_-MA-S	88	21.8
PB_30_-MA-S	88	19.8
PB_15_-CMA	91	24.8
PB_30_-CMA	93	26.8
PB_15_-RMA	89	27.8
PB_30_-RMA	87	29.8
PB_15_-RMA-S	91	18
PB_30_-RMA-S	93	30
